# Isthmin1, a secreted signaling protein, acts downstream of diverse embryonic patterning centers in development

**DOI:** 10.1007/s00441-020-03318-2

**Published:** 2020-12-28

**Authors:** Gokul Kesavan, Florian Raible, Mansi Gupta, Anja Machate, Dilara Yilmaz, Michael Brand

**Affiliations:** 1 Center for Regenerative Therapies TU Dresden (CRTD), Technische Universität Dresden, Fetscherstr 105, 01307 Dresden, Germany; 2 Max Perutz Labs, University of Vienna, A-1030 Vienna, Austria; 3 Institute for Biomechanics, ETH Zurich, Leopold-Ruzicka-Weg 4, 8093 Zurich, Switzerland

**Keywords:** Isthmin1, Isthmin2, Nodal signaling, Fgf signaling, Zebrafish, FCS, Secreted factors

## Abstract

Extracellular signals play essential roles during embryonic patterning by providing positional information in a concentration-dependent manner, and many such signals, like Wnt, fibroblast growth factor (FGF), Hedgehog (Hh), and retinoic acid, act by being secreted into the extracellular space, thereby triggering receptor-mediated responses in other cells. *Isthmin1* (*ism1*) is a secreted protein whose gene expression pattern coincides with that of early dorsal determinants, nodal ligand genes like *sqt* and *cyc*, and with *fgf8* during various phases of zebrafish development. Ism1 functions in early embryonic patterning and development are poorly understood; however, it has recently been shown to interact with nodal pathway genes to control organ asymmetry in chicken. Here, we show that misexpression of *ism1* deletion constructs disrupts embryonic patterning in zebrafish and exhibits genetic interactions with both Fgf and nodal signaling. Unlike Fgf and nodal pathway mutants, CRISPR/Cas9-engineered *ism1* mutants did not show obvious developmental defects. Further, in vivo single molecule fluorescence correlation spectroscopy (FCCS) showed that Ism1 diffuses freely in the extra-cellular space, with a diffusion coefficient similar to that of Fgf8a; however, our measurements do not support direct molecular interactions between Ism1 and either nodal ligands or Fgf8a in the developing zebrafish embryo. Together, data from gain- and loss-of-function experiments suggest that zebrafish Ism1 plays a complex role in regulating extracellular signals during early embryonic development.

**Electronic supplementary material:**

The online version of this article (10.1007/s00441-020-03318-2) contains supplementary material, which is available to authorized users.

## Introduction

Major patterning and tissue segregation events are established early during development. In zebrafish, by 6-h post fertilization (hpf), the germ layers are specified, and segregation occurs. Multiple extracellular signaling molecules play key roles in these early patterning processes, and their individual activities are carefully balanced by mechanisms such as the employment of several redundant signaling molecules for a single process and the presence of mutually antagonistic activities of different molecules. For instance, establishment of the dorso-ventral axis in zebrafish involves secreted factors from at least four signaling molecule families (Wnt, Bmp, Nodal, and Fgf), along with their respective inhibitors (Schier and Talbot, 2005). Another principle that has emerged over the last years is that besides the concerted action of agonists and antagonists, embryonic patterning in organisms also relies on the presence of specific receptors, co-receptors, and extracellular modulators that regulate the assembly of functional signaling complexes and their activity (see, e.g., Böttcher et al., 2004; Gritsman et al., 1999; Itasaki et al., 2003).

The TGFβ signaling pathway belongs to the Nodal family of proteins and includes a large number of secreted factors that are thought to signal through type I and II receptor S/T kinases with intracellular signaling via the Smads (Constam, 2014). Nodal-related genes in zebrafish such as *squint/sqt (nodal-related 1, ndr1)*, *cyclops/cyc* (*nodal-related 2, ndr2*), and *southpaw/spaw* (*spaw*) are secreted molecules that are involved in various developmental processes, including establishment of the mesendoderm and left/right asymmetry, and candidate receptors for Cyc and Sqt are the activin-like receptors ActRIIA (*acvr2aa*), ActRIIB (*acvr2ba*), and ActRIB (*acvr1ba*) (Shen, 2007; Schier, 2009). In addition to signaling via receptor S/T-kinases, Sqt and Cyc require the presence of a membrane-bound co-receptor, Egf-cfc, which is the affected molecule in the *one-eyed pinhead/oep* (t*eratocarcinoma-derived growth factor 1, tdgf1*) mutant (Gritsman et al., 1999). Further, the type I receptor TARAM-A/Tar (*acvr1ba*) has been proposed as a Nodal receptor in zebrafish because both its activated and dominant negative forms can be used to interfere with normal levels of Nodal signaling during embryogenesis (Aoki et al., 2002; Dickmeis et al., 2001; Peyriéras et al., 1998; Renucci et al., 1996).

Genes that share spatiotemporal expression profiles are likely to be involved in the same biological process (es) and are categorized into synexpression groups (Eisen et al., 1998; Niehrs and Pollet, 1999). *Isthmin* (*ism*), originally isolated in *Xenopus* and proposed as a member of the *fgf* synexpression group (Pera et al., 2002), is thought to act as an extracellular antagonist of NODAL signaling during chick development wherein it controls organ asymmetry (Osório et al., 2019). Further, Ism1 has been identified as a clefting and craniofacial patterning gene in humans (Lansdon et al., 2018) and is required for normal hematopoiesis in developing zebrafish (Berrun et al., 2018), apart from acting as an angiogenesis inhibitor and preventing tumor growth (Xiang et al., 2011). In other independent studies, Ism1 expression has been described in conjunction with transcriptome analysis of dorsal–ventral patterning genes and in a genome-wide RNA tomography study of zebrafish embryos (Bennett et al., 2007; Fodor et al., 2013; Junker et al., 2014).

Here, we describe in detail the spatiotemporal nature of zebrafish *ism1* expression and reveal that its early expression overlaps with nuclear localization of β-catenin and with the expression of *oep*, *sqt*, and *cyc*. Consistently, early expression of *ism1* depended on dorsalizing determinants such as β-catenin and Nodal signaling, whereas, in the later stages, brain-specific expression of *ism1* was under the control of Fgf signaling. In contrast to Fgf– and nodal family gene mutants, global loss-of-function Ism1 mutants, generated using CRISPR/Cas9, did not show any developmental defects. Furthermore, in vivo single molecule fluorescence cross correlation assays did not show any interaction between Ism1 and nodal ligands, suggesting that while Ism1 may not play a direct role in modulating nodal signaling pathways during early zebrafish development, it may have a complex role in regulating extracellular signals during early embryonic development.

## Results

### *Ism1* and *ism2* expression during zebrafish embryonic development

**Fig. 1:**
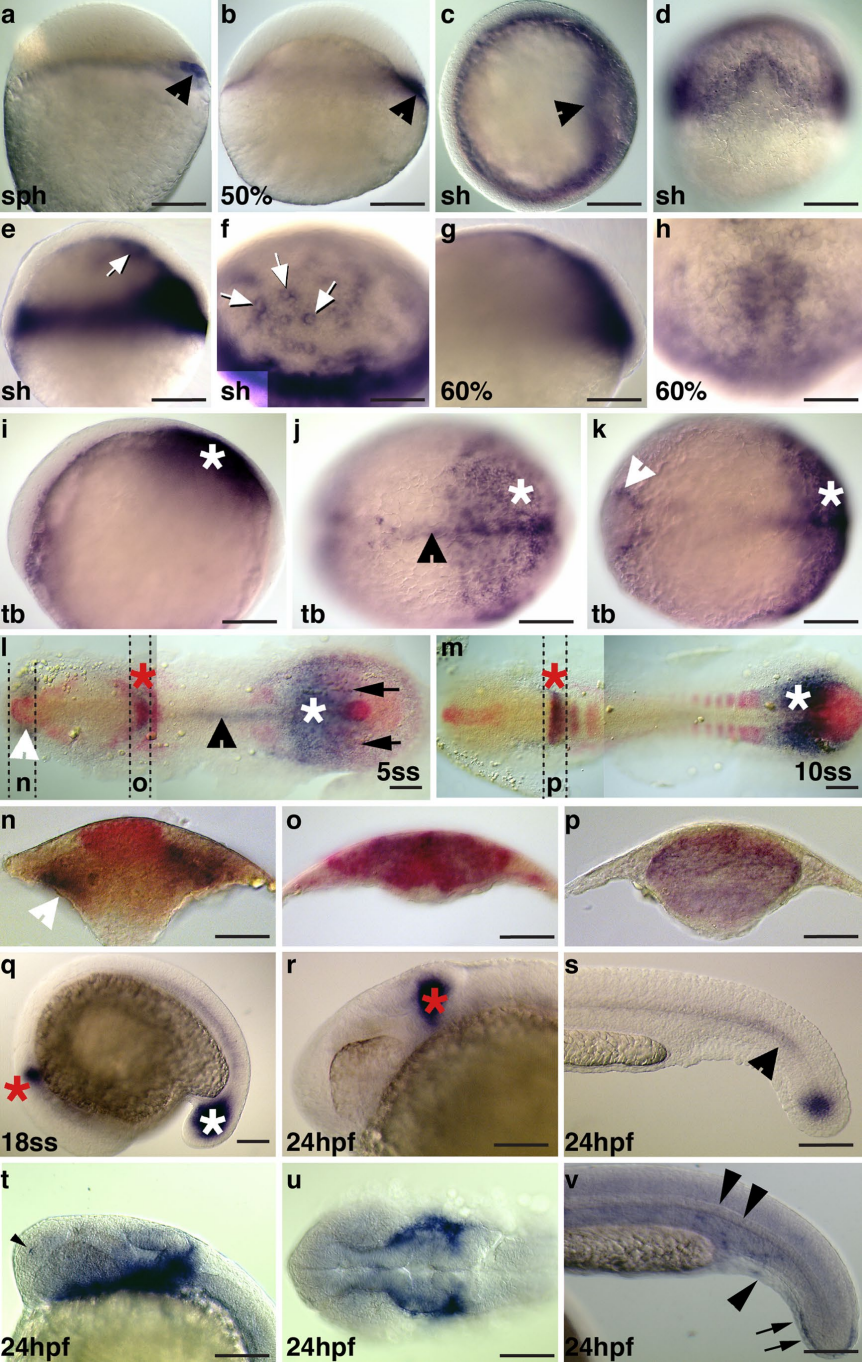
Expression of *ism1* and *ism2* during zebrafish embryogenesis. Whole mount ISH was performed at the indicated stages during zebrafish embryogenesis with riboprobes against *ism1* (panels **a**–**s**; detected in purple), *fgf8* (panels **l**–**p**, detected in red) and *ism2* (**t**–**v**; detected in purple). **a**, **b**, **e**, **g** Lateral views, dorsal to the right; **c** animal view, shield to the right; **d** corresponding dorsal view, animal to the top; **f**, **h** animal-dorsal views; **i** lateral view, anterior to the left; **j**, **k** corresponding dorsal views; **l**, **m** flat-mounted embryos, dorsal view, anterior to the left; **n**–**p** transverse sections at indicated positions in panels l and m. **q**–**t**, **v** Lateral views, anterior to the left. **u** Dorsal view, anterior to the left. Stages are indicated as % epiboly or as sph: sphere stage, sh: shield stage, tb: tailbud stage; ss: somite stage (5, 10, 18ss correspond to 5, 10, or 18 somite stage). Arrowheads in **a**, **b**, and **c** represent dorsal expression domain; arrows in **e** and **f** represent expression around YSL nuclei; white asterisks in **i** and **m** correspond to presomitic mesoderm; black arrowhead in **j** and **s** point to axial mesoderm/notochord; white arrowhead in **k** points to adaxial cells; white arrowhead in **l** and **n** corresponds to head mesenchyme; arrows in **l** point to *ism1* expression close to Kupffer’s vesicle; asterisks in **q** and **s** represent tailbud; red asterisks in **l**, **m**, **q**, and **r** correspond to the MHB; arrowhead in **t** points to expression in the nasal primodium; arrowheads in **v** represent scattered expression in the trunk; arrows in **v** point to expression in the tailfin bud. Scale bar: 200 μm in **a**–**e** and **i**–**k**; 100 μm in **f**–**h**, q, **n**–**r**, and **r**–**v**

**Fig. 2:**
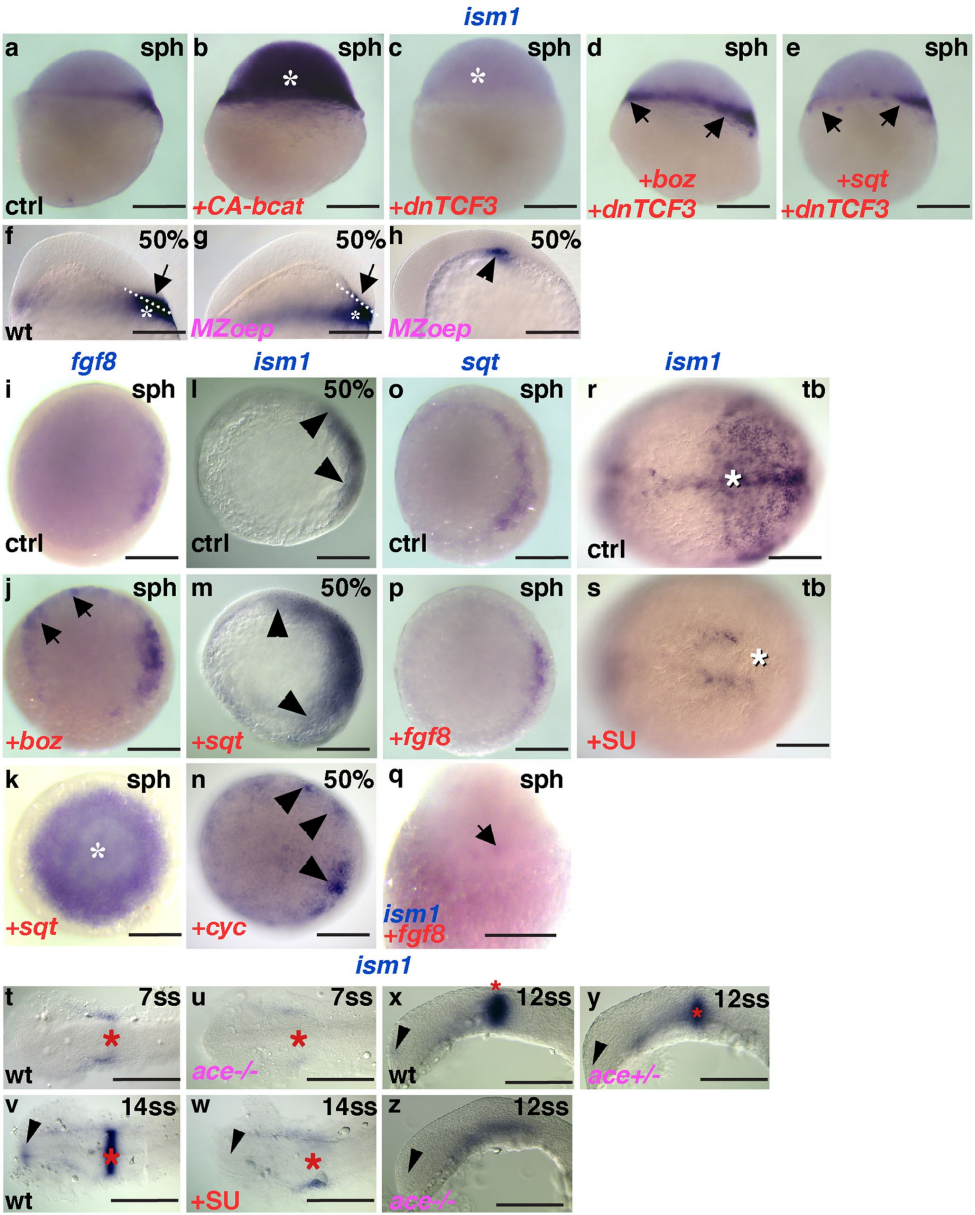
*Ism1* expression depends on dorsal determinants, and Nodal and Fgf signaling. Misexpression of constitutively active β-catenin induces ectopic *ism1* expression **b**, asterisk compared with controls **a**; **c** conversely, dominant negative TCF3 diminishes *ism1* expression; However, *boz* **d** and *sqt* **e** can induce *ism1* (arrows) in the absence of Wnt/β -catenin signaling. **f**, **g** *ism1* expression in the blastoderm (arrows) is absent in *MZoep* mutants, while its expression in the YSL remains unaffected (asterisks). **h** Residual *ism1* expression in *MZoep* mutants at the end of gastrulation (arrowhead). Both *boz* **j** and *sqt* **k** elicit ectopic *fgf8* expression (arrows, asterisk) compared with controls **i**. Both *sqt* **m** and *cyc* **n** elicit ectopic *ism1* expression compared with controls **l**. **o**–**q** Neither *sqt* **p** nor *ism1* are ectopically induced by *fgf8* overexpression. **r**–**z** Fgf-dependence of *ism1*; **r**, **s** Pharmacological FgfR inhibition during gastrulation abolishes *ism1* expression in the axial mesoderm and in the posterior paraxial mesoderm (white asterisks); **v**, **w** FgfR inhibition during somitogenesis abolishes *ism1* expression in the MHB (red asterisks) and forebrain (arrowheads); (t,u,x,z) *ism1* expression in the MHB (asterisk) and forebrain (arrowhead) is missing in *fgf8/ace* mutants. Note that expression in the MHB in panels **x**–**z** appears to depend on the genetic dose of *fgf8*. Genotypes (pink), stages (black), treatment (red), and riboprobes (blue) are indicated on the panels. **a**-**g** Lateral views, dorsal to the right; **h**, **x**–**z** lateral views, anterior to the left; **i**–**p** animal views, dorsal to the right if discernible; **q** dorsal view, animal pole to the top; **r**–**w** dorsal views, anterior to the left. Scale bar: 200 μm in **a**–**e** and **i**–**q**; 100 μm in **f**–**h** and **r**–**z**

To identify embryonic tissues in which *isthmin* may be expressed, we performed whole mount in situ hybridization with riboprobes against *ism1* and *ism2* in embryos at various developmental stages. While *ism1* could be detected early, i.e., during shield stage, tail-bud stage, and early somitogenesis, *ism2* expression could not be detected until 24-h post fertilization (hpf) (not shown). Specifically, expression of *ism1* was first detectable around the sphere stage (Fig. 1a) and was localized dorsally, based on co-expression of the organizer gene *bozozok* (*boz*)/*dharma* (*dharma*) (not shown). *Ism1* was also enriched in the dorsal embryonic margin until gastrulation (Fig. 1b). A faint expression around the marginal zone may correspond to the external yolk syncytial layer (YSL) and/or the presumptive mesendoderm. During early gastrulation, *ism1* was expressed in the anterior-most cells of the hypoblast (Fig. 1c, d). Weaker *ism1* expression found in the internal YSL at the pre-gastrula stages that became more pronounced in the shield stage (Fig. 1e, f). During gastrulation, expression in the dorsal hypoblast became restricted to predominantly axial levels (Fig. 1g, h). At the tailbud stage, expression was found in the posterior paraxial and medial aspects of the mesendoderm that also extended anteriorly to the prechordal plate (Fig. 1i–k). During early segmentation, *ism1* expression appeared in the midbrain-hindbrain region where it persisted into the later stages of embryogenesis. Prominent expression was also found in parts of the somitogenic mesoderm (Fig. 1l, m), around Kupffer’s vesicle (Fig. 2l, arrowheads), and in the notochord (Fig. 1l, m, s). During somitogenesis, co-staining for *fgf8* mRNA revealed close proximity between *fgf8* sources and domains of *ism1* expression, including in the tailbud/caudal mesoderm (Fig. 1l, m), and a strong overlap in the midbrain-hindbrain boundary region (MHB; Fig. 1o, p).

In contrast, at 24 hpf, *ism2* expression was most prominent in two bilateral streams of mesenchymal cells in the head region (Fig. 1t, u); additionally, punctate expression was observed in the tail/trunk (Fig. 1v). As the aim was to understand the role of Isthmin in early embryogenesis, we henceforth focused on only *ism1*.

Our detailed expression analysis of *ism1* during early embryogenesis in zebrafish indicates that its expression domain coincides not only with that of *fgf8*, as previously suggested (Pera et al., 2002), but also with that of early dorsal determinants and *nodal* genes such as *sqt* and *cyc* (see below). Therefore, we next investigated if either of these signaling pathways might control the expression of *ism1* or vice versa.

### Regulation of *ism1* expression during early development

The early expression of *ism1* in the dorsal aspects of the blastula suggests direct or indirect control by dorsalizing factors such as β-catenin. Additionally, nuclear localization of β-catenin in the dorsal aspects of the zebrafish YSL and in the overlying blastoderm is similar to the spatiotemporal regulation of *ism1* in the early blastula. In the presence of a constitutively active form of β-catenin (CA-β-catenin), *ism1* expression became ectopic and was visible in the entire blastula. Conversely, injection of a dominant negative variant of Tcf3 (DN-Tcf3) strongly reduced *ism1* expression (Fig. 2a–c). Together, these observations indicate that early expression of *ism1* is under the control of dorsal determinants. However, nuclear β-catenin is a transient signal and its expression, unlike that of *ism1*, may not encompass the entire blastoderm margin. Further, because Wnt signaling may extend more ventrolaterally in *Xenopus* and as β-catenin may also indirectly regulate *ism1*, we next analyzed additional factors that might regulate *ism1* expression.

The nodal signaling gene, *sqt*, and the homeobox transcription factor *bozozok/boz*, are two main targets activated by dorsal determinants. Injection of *boz* mRNA led to radial ectopic expression of *ism1* in the marginal zone of the embryos, similar to the ectopic expression of *fgf8* (Fig. 2i, j), indicating that *boz* acts upstream of *ism1*. Notably, *boz* was able to elicit *ism1* expression even when β-catenin signaling was inhibited by injecting dominant negative TCF3 (Fig. 2c, d), implying that the Wnt/β-catenin pathway may not be strictly required for *ism1* activation. Similarly, *sqt* presence was able to rescue *ism1* expression in DN-Tcf3-injected embryos (Fig. 2c, e), indicating that *sqt* is also a direct and upstream regulator of *ism1*. Notably, *sqt* expression pattern was similar to that of *ism1* as it was observed not only in the dorsal aspects of the blastula but also in the circumference of the blastoderm margin, where it is involved in specification of the mesendoderm. Likewise, *cyc* was also expressed around the blastoderm margin in the late blastula, which is consistent with a possible influence on *ism1* expression. Therefore, we next focused on a more detailed analysis of the correlation between *ism1* and Nodal signaling in the early embryo.

As the two Nodal-related ligands, Sqt and Cyc, are thought to be redundant, we analyzed *ism1* expression in maternal-zygotic *oep* (*MZoep*) mutants because Nodal signaling is thought to be completely abolished in these animals (Gritsman et al., 1999). In *MZoep* blastulae, *ism1* expression was observed in the YSL, but not in the blastoderm, indicating that *ism1* expression in the YSL is independent of Nodal signals (Fig. 2f, g). During gastrulation, *ism1* transcripts were not detectable in *MZoep* mutants except in the YSL, which is consistent with the fact that *MZoep* embryos lack most of the mesendoderm. However, at the tailbud stage, we noticed a small deep-layer expression domain in the dorsal side of the embryo, which may represent residual ventral mesendoderm after its convergence with the dorsal side of the embryo (Fig. 2h). Consistent with this interpretation, *lefty1*/*antivin* (*lft1*)-injected embryos that lack the mesendoderm did not display residual *ism1* expression (data not shown).

Conversely, injection of both *sqt* and *cyc* mRNA elicited ectopic *ism1* expression in the blastula (Fig. 2l–n), indicating that Nodal signaling is not only necessary for early *ism1* expression but also that it is sufficient to ectopically induce *ism1* expression. In contrast to these early-stage observations, later-stage *ism1* expression appeared to be independent of Nodal signaling. Specifically, the onset of ectodermal *ism1* expression in the MHB was not affected in *MZoep* embryos, and its expression in this region persisted even in the later stages, i.e., in the telencephalon as well (data not shown).

The overexpression of *sqt* induced the ubiquitous expression of *fgf8* in the sphere stage (Fig. 2i, k), raising the possibility that *fgf8* might mediate the induction of *ism1* by *sqt*. Therefore, we investigated *ism1* expression during perturbed Fgf signaling, wherein FgfR-dependent signaling was inhibited between fertilization and the late blastula stage using the small molecule inhibitor SU5402 (Reifers et al., 2000). This did not lead to a significant decrease in *ism1* expression (not shown), and similar results were obtained after the injection of RNA encoding a dominant negative Fgf receptor (Hongo et al., 1999) at the 1-cell stage (data not shown). Moreover, we could not detect any changes in *ism1* or *sqt* expression at the sphere stage in *fgf8* mRNA-injected embryos (Fig. 2o–q). These results imply that the earliest expression of *ism1* is not controlled by Fgf signaling.

In contrast, when SU5402 was added to the medium during the late gastrulation stages, *ism1* expression was predominantly abolished (Fig. 2r, s); this pattern of change in gene expression is similar to that seen with other target genes of the Fgf signaling pathway, such as *pea3*, whose expression was used to ensure the efficiency of inhibitor treatment. Moreover, *ism1* transcripts were completely absent from the MHB when embryos were treated with SU5402 from early to mid-somitogenesis (Fig. 2v, w), indicating that subsequent neuroectodermal expression of *ism1* depends on FgfR signaling. A major Fgf ligand involved in MHB organization is Fgf8. Consistent with a role for Fgf8 in the regulation of *ism1* expression in the MHB, *ism1* expression was absent in homozygous *fgf8/ace* mutant fish and it was reduced in heterozygous mutants (Brand et al., 1996; Reifers et al., 1998) (Fig. 2t, u, x–z).

**Fig. 3:**
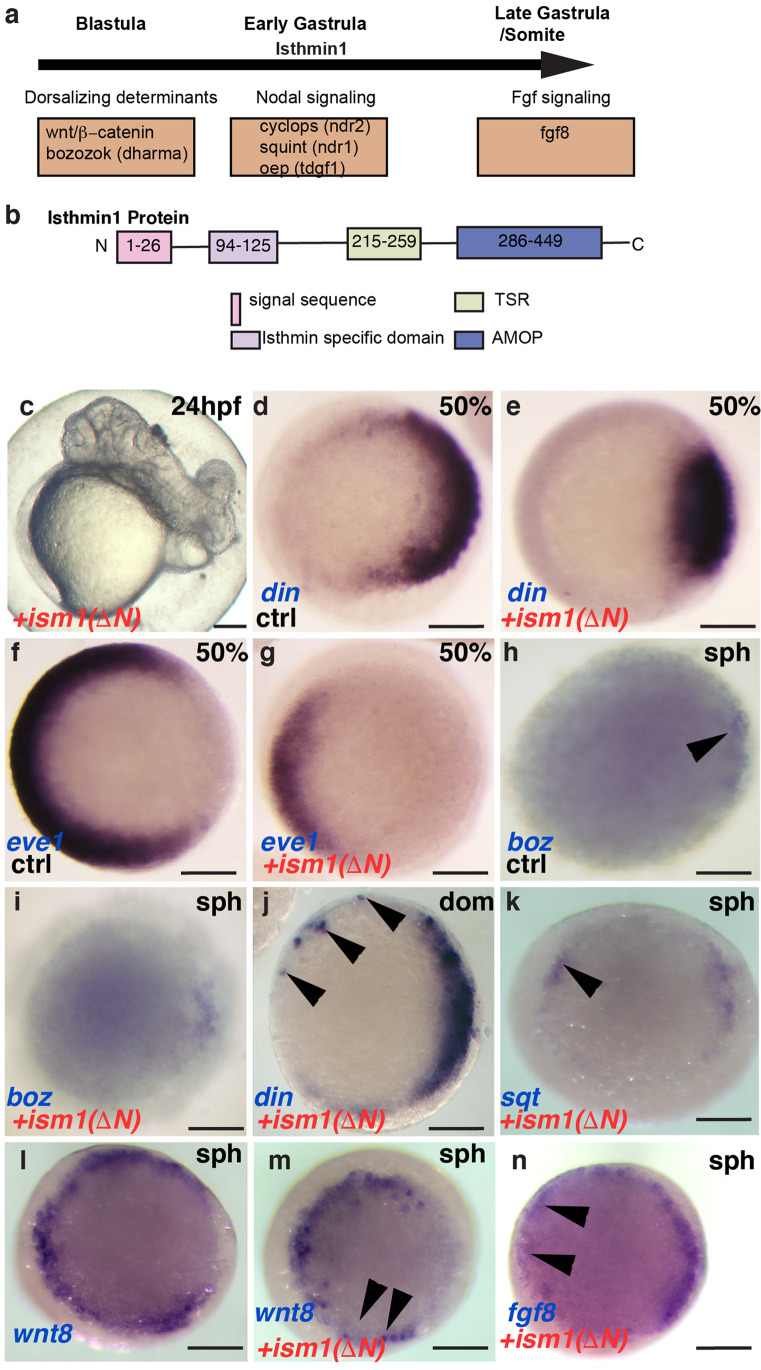
Dorsalization of embryos in *ism1* deletion mutants. **a** Schematic representation of upstream regulators of *ism1* during various stages of embryonic development. **b** Schematic representation of the Ism1 protein and the amino acid length of its domains, signal sequence, Isthmin specific domain (ISD), thrombospondin-type 1 repeat (TSR), and an adhesion-associated domain in Muc4 and other proteins (AMOP). **c** Dorsalized and cyclopic phenotype observed in ism1 (∆*N*) mRNA-injected embryos at 24 hpf (see Table 1). **d**–**g** Dorsalization of the injected embryos at 50% epiboly, or late blastula (see Table 2). **d**, **f** Uninjected controls and **e**, **g** ism1(∆*N*)-injected embryos were probed for *din* and *eve1* as dorsal and ventral markers, respectively. **h**, **i** *boz* appears unchanged after injection of ism1 (∆*N*). **j**, **k**, **n** *din*, *sqt*, and *fgf8* can be induced by ism1 (∆*N*) ectopically (arrowheads); **l**, **m** wnt8 (ORF1 + ORF2) is downregulated in the marginal zone. Left is ventral, and right is dorsal in all pictures. **d**–**n** Animal pole views. The time of color development is shortened in **k** to emphasize the ectopic up-regulation of *sqt* expression. Scale bar: 200 μm

Taken together, these results imply that *ism1* expression is temporally controlled by different signaling systems (Fig. 3a): while its early expression is controlled by dorsal determinants such as *boz* and *sqt*, blastoderm expression requires nodal signaling, and its later expression, especially in the midbrain-hindbrain boundary, is responsive to Fgf8 levels.

### Dorsalization of embryos in Ism1 deletion variants

Ism1 is characterized by the presence of multiple sequences such as the thrombospondin-type 1 repeat (TSR), an adhesion-associated domain in Muc4 and other proteins (AMOP) sequence, a signal peptide sequence, and a conserved sequence present in all Isthmins, which we refer to as the Ism-specific domain (ISD) (Fig. 3b). To dissect the function of *ism1* and these three protein motifs, we designed expression constructs with full-length *ism1* and deletion variants and injected in vitro transcribed mRNA of these constructs into fertilized zebrafish eggs to verify if they caused patterning abnormalities.

Consistent with earlier observations (Pera et al., 2002), full-length *ism1* did not appear to cause strong morphological defects. However, a construct that lacked the N-terminal portion including the IND-motif but retained the signal peptide, *ism1* (∆*N*), caused dorsalization of 23% of the injected embryos, and cyclopia, albeit with lower frequency (Fig. 3c; Table 1). When we removed the remaining C-terminal portion of the molecule, we found that both the TSR and AMOP domains elicited a similar phenotype on their own and with similar penetrance (Ism1-TSR and Ism1-AMOP; summarized in Table 1), implying that both domains contribute to the effects observed with *ism1* lacking the ISD motif. In contrast to the marked dorsalization and cyclopia, none of the deletion constructs disturbed the general morphology of the isthmic constriction, whose formation critically depends on functional Fgf signaling (Reifers et al., 2000; Meyers et al., 1998). As dorsalization was the most prominently observed phenotype, we next investigated if this early dorso-ventral polarity was disturbed in embryos injected with Ism1 deletion variants. Using *din* (*chordin*, *chrd*) as a dorsal-specific marker (Miller-Bertoglio et al., 1997) and *evenskipped/eve1* (*eve1*) as a ventral marker (Joly et al., 1993), we found that embryos injected with *ism1* (∆*N*), *ism1-TSR*, or *ism1-AMOP* were dorsalized at the late blastula stage (Fig. 3d–g; Table 2). Further, as *Sqt* and *boz* act upstream of *din* while establishing the dorsal organizer in zebrafish, we tested if ectopic activation of *din* was preceded by ectopic expression of *sqt* or *boz*. Upon misexpression of *ism1* (∆*N*), punctuate expression of *sqt* could be detected at the sphere stage itself; however, we did not observe ectopic *boz* expression, suggesting that the expansion of *din* could be mediated by ectopic activation of *sqt* alone (Fig. 3h–k). Consistent with the observed upregulation of *sqt* and *din*, we noted that *wnt8* expression pattern was interrupted in injected embryos, indicating that ectopic activation of organizer-genes occurred at the expense of ventrolateral germ ring identity (Fig. 3l, m). Further, ectopic expression of fgf8 could be induced by ism1 (∆N) (Fig. 3n). These data are consistent with the notion that early dorsalization of the embryo upon *ism1* (∆*N*) injection is due to overactivation of the Nodal signaling pathway. Further, animals injected with *Ism1*-∆N-RFP (c-terminus fusion construct) or the untagged *Ism1-∆N* were indistinguishable in terms of the dorsalization phenotype (data not shown).

**Table 1:** Phenotypes elicited by ism1 deletion constructs after 24 h of development

Construct	Total	Normal	Dorsalized	Cyclopic eye	Dorsalized + Ectopic eye
*ism1*	228	221 (97%)	7 (3%)	0	0
*ism1-N*	162	162 (100%)	0	0	0
*ism1(ΔAMOP)*	168	168 (100%)	0	0	0
*ism1(ΔTSR)*	178	178 (100%)	0	0	0
*ism1(ΔN)*	198	127 (69%)	45 (23%)	4 (2%)	12 (6%)
*ism1-TSR*	170	104 (62%)	51 (30%)	4 (2%)	11 (6%)
*ism1-AMOP*	171	117 (68%)	46 (27%)	0	8 (5%)
s*GFP*	92	92 (100%)	0	0	0

**Table 2:** Dorsalization elicited by ism1 deletion constructs at shield stage

Construct	Total	Normal	Dorsalized
*ism1*	126	122 (97%)	4 (3%)
*ism1-N*	133	133 (100%)	0
*ism1(ΔN)*	173	113 (65%)	60 (35%)
*ism1-TSR*	122	94 (77%)	28 (23%)
*ism1-AMOP*	109	82 (75%)	27 (25%)

In contrast to the Ism1 versions lacking the N-terminal ISD domain, the mRNA for all versions of Ism1 contain the N-terminal half of Ism1, including wild-type Ism1, caused little, or no dorsalization in assays (Table 2), implying that the N-terminal half of Ism1 was responsible for the observed dorsalization.

### Generation and characterization of *ism1* mutants

**Fig. 4:**
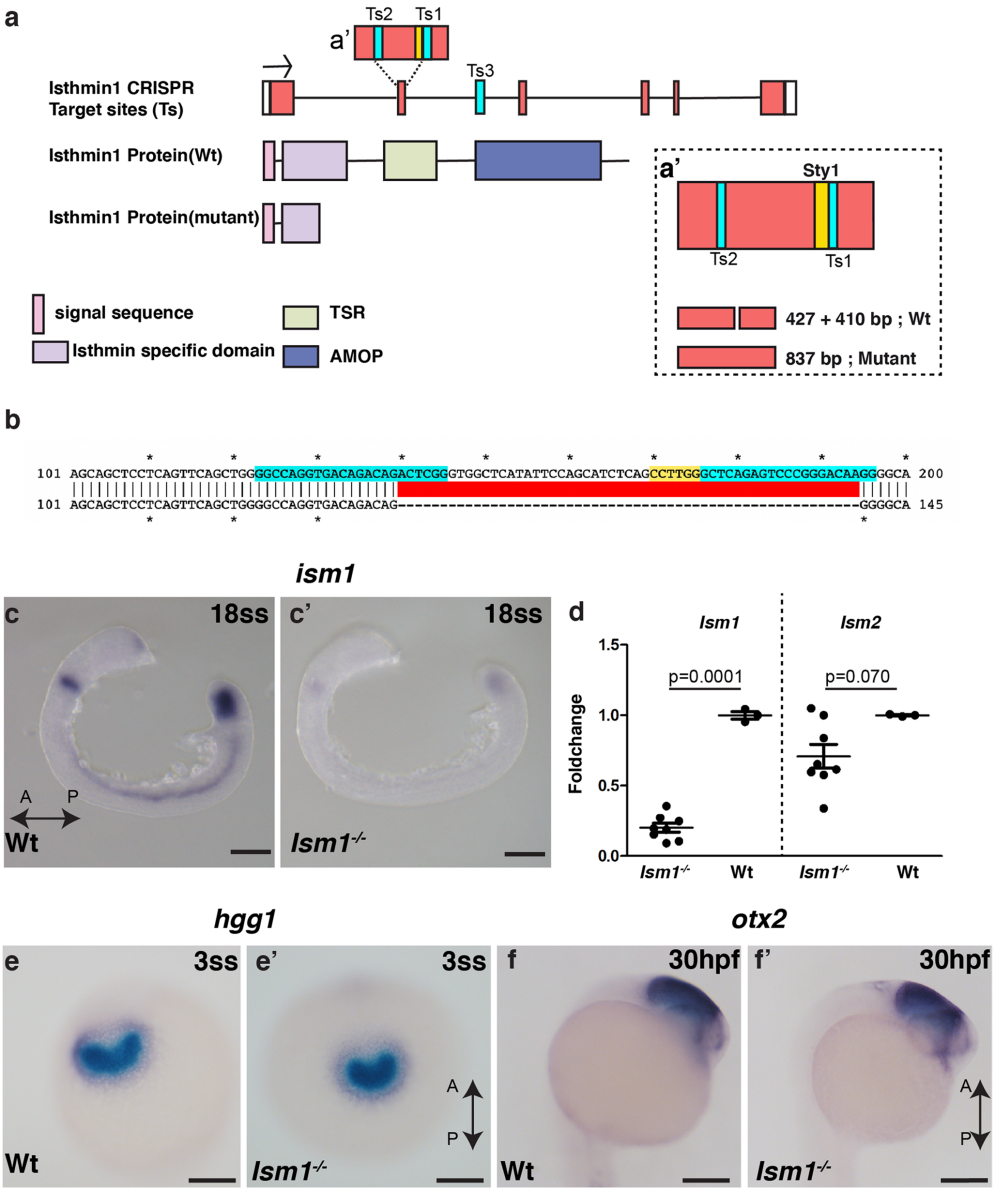
Generation and characterization of *ism1* mutants **a** CRISPR gRNA target sites (Ts) were located in the exon (Ts1 and Ts2) and in the intron (Ts3). The sty1 restriction site is marked in yellow. Scheme representing protein domains in wild-type Ism1 and in the truncated version in CRISPR mutants are shown. Schematic representation of the genotyping strategy shown in **a′**, loss of the Sty1 restriction site in the mutants will result in an 837-bp band, while in the Wt there will be two bands (i.e., 437 + 410). **b** Sequence alignment between Wt and Ism1 mutant embryos shows 55 bp deletion, in cyan (Ts), and yellow (Sty1 restriction site). **c** and **c′ **Whole mount in situ hybridization in 18 somite stage (18ss; 18 hpf) embryos for Ism1 shows decreased expression in Ism1−/− embryos compared with the wild type (Wt) embryos. **d** qRT PCR for *Ism1* and *Ism2* in 24 hpf embryos of *ism1*^*−/−*^ mutants show a significant reduction in only *Ism1* mRNA with no change in *Ism2* levels. **e** and **e′** Whole mount in situ hybridization for *hgg1*, a marker for the anterior prechordal plate and the later hatching gland, shows similar expression and distribution patterns in the hatching gland between Wt and Ism1−/− embryos. Ventral images are shown. **f** and **f′** Midbrain marker *otx2* shows no obvious difference between Wt and Ism1−/− embryos. The anterior–posterior axis of the embryo is marked as A-P with an arrow. The total number of embryos **n** analyzed in panels c (*n* = 20), d (*ism1*^*−/−*^: *n* = 8, Wt: *n* = 3), e (*n* = 40), and f (*n* = 40). *P* values from unpaired *t* tests are indicated within the graph **d**. Scale bar 100 μm

To assess if Ism1 was required for embryonic development, we used CRISPR-Cas9 mediated mutagenesis to create stable mutant lines. Targeting two sgRNAs (Ts1 and Ts2) in the Ism-specific domain, ISD, resulted in multiple mutant alleles. We selected a 55-bp deletion and established founder lines for this mutation (Fig. 4a, b). This deletion is predicted to yield a truncated protein (91 instead of 461 amino acids) wherein the first 91 amino acids span the signal peptide, parts of the ISD domain (Fig. 4a) and terminate before the TSR and AMOP domains. Founders (F0) were out crossed to wild types (F1), and the mutant alleles were maintained as outcrosses. Mutants were identified by PCR, and the concomitant loss of a *StyI* restriction site was used to differentiate wild-type and mutant alleles (Fig. 4a′). In situ hybridization using an *ism1* riboprobe yielded only faint *ism1* mRNA staining in *ism1* mutants, consistent with the notion that mutant *ism1* mRNA, with its premature stop codon, undergoes nonsense-mediated decay (Fig. 4c, c′). Next, qRT PCR for *Ism1* and *Ism2* in 24 hpf embryos of *ism1* mutants showed a significant reduction in only *Ism1* mRNA with no change in *Ism2* levels (Fig. 4d). Homozygous and heterozygous mutants generated by F1 in-crosses (F2) showed no developmental abnormalities and could be raised to adulthood, indicating that the loss of *ism1* can be compensated for by other molecules during development. Next, we analyzed multiple markers during mes-endoderm differentiation and in the Nodal pathway but found no observable phenotypic differences between wild-type and homozygotic mutants. The hatching gland, marked by the expression of *hgg1*, which is derived from the pre chordal plate mesoderm and the anterior axial mesoderm, also showed no observable differences between the wild-type animals and *ism1* mutants (Fig. 4e, e′). In *oep* mutants, *hgg1* expression is strongly reduced or absent. Additionally, analysis of *otx2*, a marker for anterior neuroectoderm and later for the midbrain, showed a similar expression pattern in the wild-type and *ism1* mutants, suggesting no defects in head formation observed in various mutants of the nodal pathway (Fig. 4f, f′). To explore whether the lack of developmental phenotypes was due to the specific allele generated, an independent sgRNA (Ts3) targeting a site within an intron downstream of the ISD was injected along with Ts1 and Ts2. The resulting 2.5-kb deletion mutant allele did not exhibit any developmental phenotype either (data not shown). Taken together, these loss-of-function results indicate that *ism1* is dispensable during early embryonic development in zebrafish.

### Ism1 diffuses in the extracellular space, but does not directly interact with Fgf or Nodal ligands

**Fig. 5:**
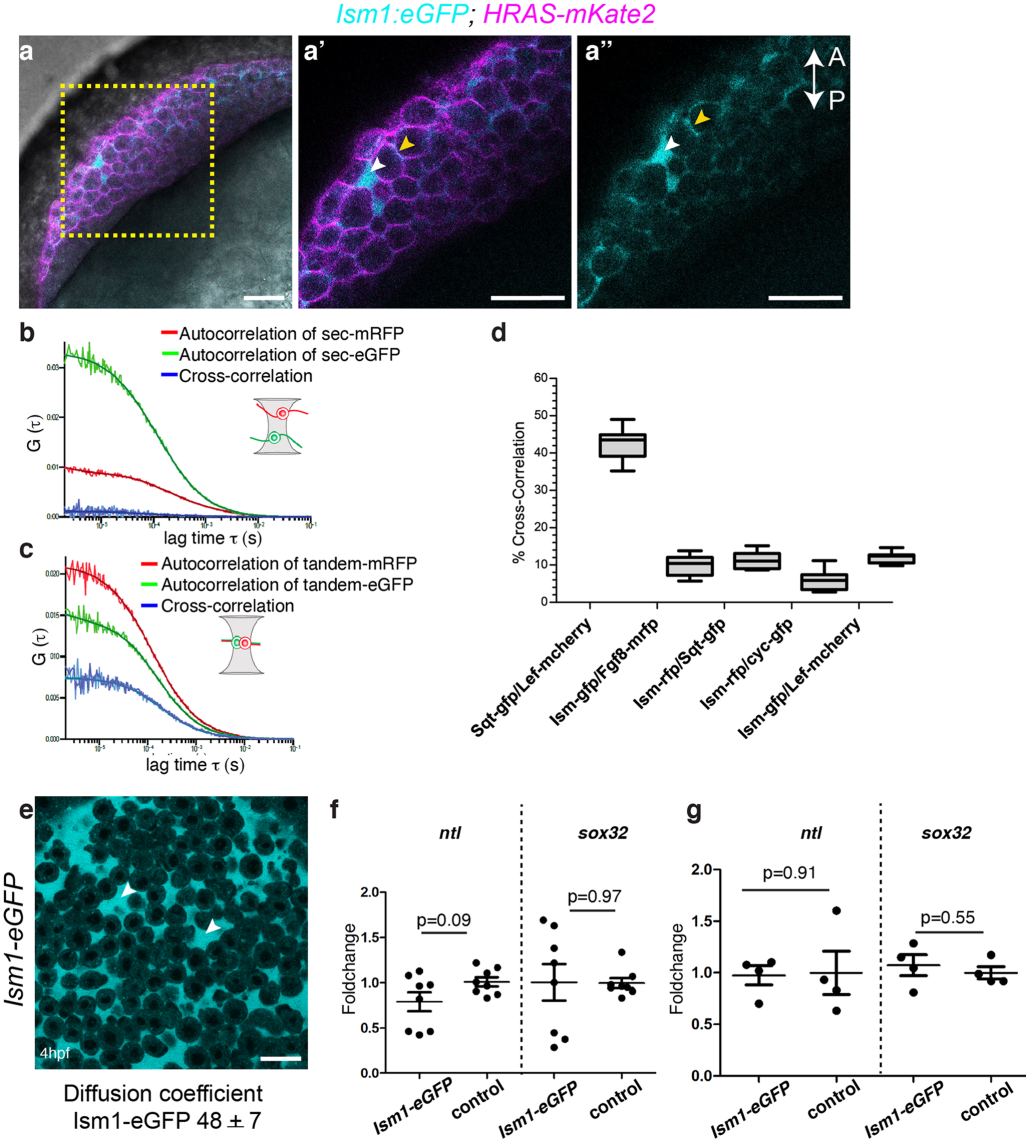
Ism1 interaction with Fgf8 and Nodal molecules in vivo **a** *Ism1-eGFP* and membrane localized *mKate2* (*HRAS-mKate2*) was co-injected at 1-cell stage embryo, and images from live embryos were obtained at 50% epiboly (5.3 hpf). *Ism1-eGFP* was predominantly observed in the extra cellular space (white arrowhead) and close to the plasma membrane (yellow arrowhead). **a′** and** a″** Magnified images of the marked region in **a**. **b**, **c** By cross-correlating fluorescence fluctuations (FCCS) in two spectral channels, bimolecular binding can be inferred because only co-diffusing binding partners lead to a considerable cross-correlation. Coinjection of secreted mRFP (Sec-mRFP) and secreted eGFP (Sec-eGFP) showed no cross- correlation, while a tandem construct with mRFP and eGFP showed cross- correlation. **d**. sqt and lefty have about 45% cross correlation, while that between Ism1 and fgf8a, sqt, cyc, or lefty was about 15%, which is in the range of random interactions (background). **e** The diffusion coefficient of *Ism1-eGFP* was measured using FCS in the extracellular space (indicated with arrowhead) of sphere stage embryos (4 hpf) injected with *Ism1-eGFP* mRNA at the 1-ll stage. *Ism1-eGFP* diffusion coefficient was 48 ± 7 μm^2^ s^−1^ **f **and **g**. qRT PCR analysis of Ism1-eGFP or mCerulean (control) injected embryos (mRNA or DNA) showed no significant difference in the gene expression levels of the mesoderm marker *ntl* or the endoderm marker *sox32*. Embryos were harvested at 50% epiboly (5.3 hpf), and each sample represents a pool of 10 embryos. *P* values from unpaired *t* tests are indicated within the graph. Numbers of embryos analyzed for FCS and FCCS are presented in Table 3. The total number of embryos (n) analyzed in panel a (*n* = 8). Scale bar 20 μm

To test whether Ism1 is a secreted protein, we created an expression construct that contained the full-length Isthmin with a C-terminally fused GFP moiety (Ism1-GFP) and co-injected this with a membrane-bound mKate2 fluorescent protein (HRAS-mKate2) into one-cell-stage embryos. This resulted in a fluorescent signal at or close to the plasma membrane and in the extracellular space (Fig. 5a, a′, and a″, arrowhead), supporting the idea that Ism1 is a secreted protein. Further, overexpression of full-length Ism1-GFP did not lead to any obvious dorsalization phenotype and these animals were similar to those injected with the untagged full-length protein (Supplementary Fig. S1a-b).

Despite the dispensability of *ism1* during early embryonic development, our results suggested a possible interaction between Ism1 and Fgf or Nodal signaling components. To assess if Ism1 interacts in vivo with components of either Fgf or Nodal pathways and to possibly modify their respective activities during development, we utilized fluorescence correlation spectroscopy (FCS), wherein interaction between biomolecules is detected through their correlated motion in space and time (Ries et al., 2009; Wang et al., 2016; Yu et al., 2009). FCS is based on detecting fluorescence fluctuations in a small confocal volume (∼ 0.5 fl). Statistical analysis by autocorrelation of these fluctuations provides quantitative information on local concentrations and diffusion coefficients of the fluorescent molecules present. By cross-correlating fluorescence (FCCS) fluctuations in two spectral channels, bimolecular binding can be inferred, because only co-diffusing binding partners will lead to a considerable cross-correlation. In other words, a strong cross-correlation percentage indicates a high probability of detecting both fluorescent molecules in the observed volume due to the bimolecular interactions.

Embryos that were coinjected with secreted forms of mRFP (Sec-mRFP) and eGFP (Sec-eGFP) showed no cross-correlation (negative control), while a control tandem construct with mRFP and eGFP showed cross-correlation (positive control) (Fig. 5b, c). Moreover, a test employing tagged Squint and Lefty proteins (Sqt-gfp, Lef-mcherry) showed significant cross-correlation of these two factors (45%) (Fig. 5d), as previously shown (Wang et al., 2016). These results established that this FCCS system can be used to infer binding partners in vivo.

Full length Ism1-GFP fusion mRNA was injected into 1-cell stage embryos, and tagged ligands of Fgf and Nodal pathways, namely, Fgf8-mcherry, Sqt-RFP, Cyc-RFP, and Lefty-RFP, were injected at the 32-cell stage. Dual color FCCS was performed to evaluate potential interactions between Ism1 and Fgf8 or the Nodal ligands. Ism1-eGFP and Fgf8a-mRFP fusion proteins showed weak cross-correlation percentage (14% compared with 45% for Sqt-lft). Similarly, Ism1-rfp showed low cross-correlation percentage with nodal ligands sqt and cyc and the nodal antagonist lft (14%, 10%, and 15% respectively, compared with 45% for Sqt-lft). These FCCS data did not reveal strong interactions between ism1 and Fgf8a, or any of the nodal signaling molecules tested (Table 3; Fig. 5d). Next, to investigate how Ism1 diffuses in the extracellular space, FCS was carried out at the animal pole in sphere stage embryos where ectopically expressed *ism1-eGFP* was the only source of *ism1*. The FCS autocorrelation curve fitted well to a three-dimensional diffusion model with a diffusion coefficient of 48 ± 7 μm^2^ s^−1^, and 100% of the molecules were fast diffusing. Taken together, these results indicate that Ism1 freely diffuses in the extracellular space, but that there is no strong interaction with Nodal components or Fgf8 (Fig. 5e).

**Table 3:** FCS and FCCS sample size

Molecules	Cross correlation (%)	No of embryos (*n*), Total no of readings (*R*)
*Squint-gfp vs lefty-mcherry*	45	*n* = 3, *R* = 3
*isthmin-gfp vs Fgf8-mrfp*	14	*n* = 3, *R* = 11
*Ism-rfp vs Squint-gfp*	14	*n* = 8, *R* = 10
*Ism-rfp vs Cyc-gfp*	10	*n* = 5, *R* = 13
*Ism-gfp vs lefty-mcherry*	15	*n* = 8, *R* = 12
*Ism-gfp*	FCS for diffusion coefficient	*n* = 11, *R* = 34

Next, to address if overexpression of full length Ism1 (gain-of-function) has an impact on Nodal signaling, we used mes-endoderm differentiation as the read-out as this process is a consequence of active Nodal signal transduction. Ism1-GFP mRNA or mCerulean was injected at the 1-cell stage, embryos were fixed at 50% epiboly (5.3 hpf), and in situ hybridization was used to evaluate markers belonging to the mes-endoderm lineage, namely, *ntl* for the mesoderm and *Sox32* for the endoderm. However, ISH pattern and signal intensity were indistinguishable between the gain-of-function mutants and wild-type embryos, and these results were corroborated using quantitative RT-PCR, suggesting that Ism1 may not affect nodal signaling during embryonic development in zebrafish (Fig. 5f, g). Thus, even though Ism1 diffuses freely in the extracellular space, it does not directly interact with Fgf or Nodal ligands, and the overexpression of full length Ism1 does not alter mesendoderm differentiation.

## Discussion

Secreted proteins have the potential to influence multiple signaling cascades, for example, secreted morphogens, such as Fgf and Wnt control patterning and development in a context- and concentration-dependent manner (Bökel and Brand, 2013).

While the gene expression pattern of Ism1 was interesting and suggested possible association with sites of Nodal activity, such as the dorsal hypoblast, the YSL, the mesendodermal layer of the gastrula, and the prechordal plate, loss-of-function, misexpression experiments with Ism1 deletion constructs yielded contrasting results. Nonetheless, misexpression of an Ism1 derivative without the ISD-containing N-terminus, Ism1(∆*N*), caused marked dorsalization of the blastula and, *sqt* and other genes characteristic of the dorsal blastula, were ectopically induced in the presumptive mesendoderm. Such dorsalization and ectopic induction of *nodal* genes are known to be caused by the ectopic activation of Nodal signaling (Erter et al., 1998; Rebagliati et al., 1998; Feldman et al., 1998; Shimizu et al., 2000). In contrast, loss-of-function Ism1 mutants did not show any developmental defects, suggesting complex molecular interactions of Ism1 (∆*N*) in the early blastula stage embryos. Our results with the Ism1 (∆*N*) deletion constructs, namely, dorsalization, and nodal signaling, are in agreement with those from a recent study in chick, wherein Ism1 was shown to interact with a Nodal ligand and type 1 receptor ACVR 1B to influence organ asymmetry (Osório et al., 2019). However, observations with full length Ism1 in chicken could not be observed in zebrafish and this could be due to sequence differences downstream of the signal peptide (Osório et al., 2014) (Supplementary Fig. S1c). Additionally, ISH and qRT-PCR analysis with full length Ism1 overexpression did not reveal problems in mesendoderm differentiation, suggesting differences in molecular interaction between full length Ism1 and the Ism1 (∆*N*) protein.

Loss-of-function experiments with morpholinos targeting the 5′UTR and the start codon to prevent Ism1 translation in zebrafish embryos result in phenotypes affecting the hematopoietic stem and progenitor cells (HSPCs), rather than abnormalities or phenotypes resembling known nodal pathway mutants (Berrun et al., 2018). Similarly, Xiang et al. (2011) have shown that isthmin1 may be an angiogenesis inhibitor, and knocking down isthmin-1 with morpholino targeting the splicing site yielded no phenotype affecting early embryonic patterning or morphogenesis (Xiang et al., 2011). Although disorganized inter-segmental vessels were observed in the study by Xiang et al., we did not test such phenotypes in our mutant lines. Importantly, these results using morpholinos are in line with our observations with CRIPSR mutants which also showed no observable early embryonic patterning phenotype. Furthermore, it is possible that the loss of Ism1 in the early embryos could be compensated for by the presence/activity of other genes, leading to the absence of a noticeable phenotype or by the phenomenon of genetic compensation triggered by mutant mRNA (El-Brolosy et al., 2019; Rossi et al., 2015).

The diffusion coefficient of Ism1 (48 ± 7 μm^2^ s^−1^) is comparable to that of Fgf8a (53 ± 7 μm^2^ s^−1^), suggesting free diffusion in extracellular space; however, in contrast to Fgf8 (Yu et al., 2009), there was no slow diffusing fraction in Ism1. The slow diffusing fraction of Fgf8 is due to its interactions with heparin sulphate proteoglycans (HSPG) in the ECM, but Ism1 may not be restricted by such molecules in the extracellular space. While these results from FCS and FCCS cannot exclude the possibility of Ism1 interacting with other signaling pathways, the complex temporal regulation of *ism1* argues for a more intricate role for this factor, maybe as a multivalent cofactor in extracellular signaling. Furthermore, shared molecular motifs and domains on Ism1 also argue for the presence of such a crosstalk between signaling pathways. For instance, R-Spondin2, a novel secreted activator of Wnt/β-catenin signaling, also possesses a TSP1 domain that does not seem to be necessary for Wnt-related activity (Kazanskaya et al., 2004). Moreover, *ism1* is also expressed in the midbrain-hindbrain area, where its expression is critically dependend on FgfR-signaling. As the midbrain-hindbrain boundary is a known source of extracellular signaling molecules of the Fgf and Wnt class (reviewed in Raible and Brand, 2004; Gibbs et al., 2017), our results support a possible role for Ism1 in multiple signaling processes, and the presence of viable adult Ism1 mutants can shed light on its role in tissue homeostasis and regeneration.

In summary, we describe in detail the expression profile of *ism1* during development and its upstream activators during various stages of embryonic development in zebrafish. Further, dorsalization phenotypes with the deletion mutants support a role for *ism1* in early patterning, while no developmental defects with the CRISPR mutants suggests that Ism1 is dispensable or its lack compensated for, during early embryonic development.

## Materials and methods

### Zebrafish maintenance and mutant strains

Zebrafish were maintained and staged according to standard conditions (Brand et al. 2002; Westerfield, 1994). Mutant lines used in this study were *one-eyed pinhead/oep*^*z1*^ (Schier et al., 1996; Solnica-Krezel et al., 1996), *squint/sqt*^*cz35*^ (Heisenberg and Nüsslein-Volhard, 1997), and *acerebellar/ace*^*ti282a*^ (Brand et al., 1996; Reifers et al., 1998).

### In situ hybridization

Whole mount in situ hybridization was performed as previously described (Reifers et al., 1998), and tissue sections were obtained using tungsten needles. Plasmids used for in situ hybridizations were obtained from the following sources: *cyc*, M. Rebagliati; *din*, M. E. Halpern; *eve1*, M. Hammerschmidt; *hgg1*, C.-P. Heisenberg; *lefty2*, J. Bischof and W. Driever; *sox17*, D. Stainier; and *wnt8*, A. Lekven.

### DNA constructs and injections

The full *ism1* coding sequence was identified by a screen for Fgf-dependent genes (our unpublished data). A partial *ism2* cDNA fragment was isolated by RT-PCR using cDNA from 24 hpf embryos, and the fragment was extended using inverse PCR and 5′RACE.

cDNAs for *ism1*, *ism1:eGFP*, *sqt*, and *fgf8* were subcloned into pCS2 + vectors for in vitro transcription. The sources of other plasmids used for mRNA injection are as follows: *lefty1*/*antivin*, K. Knight and J. Yost; ∆*Xfgfr4a*, H. Okamoto; and dnTCF3 and constitutively active β-catenin: T. Hirano. The dominant negative Tcf3 (dnTcf3) lacks the beta-catenin binding domain and has been shown to abrogate both beta-catenin translocation to the nucleus and beta-catenin-mediated transcriptional activation (Molenaar et al., 1996). Capped mRNA for injection was transcribed from linearized plasmid templates using the SP6 mMESSAGE mMACHINE kit (Ambion). For zebrafish injections, mRNA was injected at a default concentration of 100–200 pg/embryo in the one-cell stage.

### Pharmacological treatments

To block embryonic FgfR signaling, embryos were incubated with diluted SU5402 (Calbiochem/EMD Biosciences) in embryonic medium E3 at a final concentration of 16 µM as described (Reifers et al., 2000).

### Confocal microscopy

*Ism1-eGFP* mRNA-injected embryos were mounted in a drop of 1.5% low-melting temperature agarose in E3 buffer, and fluorescence was observed using a laser scanning microscope.

### CRISPR-Cas9 mutagenesis

Two target sites (Ts1 and Ts2) in Ism1 cDNA were identified using the webtool chopchop (Montague et al., 2014). Both SgRNA and Cas9 mRNA were prepared as previously described (Jao et al., 2013). Cas9 mRNA was injected at 150 ng/µl, and Ts1 and Ts2 sgRNA were injected at 20 and 35 ng/µl, respectively. All injections were carried out in the wildtype AB strain, and injected embryos were raised and outcrossed with wild-type strains (AB). Founders were identified by PCR and restriction digestion of F1 embryos which was later also confirmed by DNA sequencing. Those mutations that predicted a premature termination of the protein were maintained as heterozygotes. For the 2.5-kb deletion mutant, all three sgRNA (Ts1-3) were coinjected at a concentration of 20 ng/µl each. The sequence for Ts1-3 are provided in Table 4.

**Table 4:** *Ism1* SgRNA sequence of the target sites, PAM sequence is underlined

Target site (Ts)	Sequence
Ts1	GGGCTCAGAGTCCCGGGACAAGG
Ts2	GGCCAGGTGACAGACAGACTCGG
Ts3	GGTCATGGTCATGCACCACGAGG

### Genotyping

Genomic DNA was extracted either from single embryos (1 day old) or from finclip samples (3 months old) (hot shot DNA extraction method) or from PFA fixed embryos after in situ hybridization (Yang and Gu, 2017). PCR was performed using primers that flank the target site; the primer sequences are provided in Table 6. PCR products were digested with the restriction enzyme Sty1 and analyzed by gel electrophoresis wherein wild types produced 427- and 410-bp bands, while mutants produced one 837-bp band. All genotyping results were later confirmed by sequencing.

### Fluorescence correlation spectroscopy (FCS) and Fluorescence cross correlation spectroscopy (FCCS)

Messenger RNA coding Ism1 eGFP or Ism1 RFP was injected into the cytoplasm of one-cell stage embryos, and various nodal components were injected into one cell of a 32-cell-stage embryo expressing Ism1 eGFP or Ism1 RFP. FCS and FCCS were carried in embryos at the sphere and dome stage (4 and 4.3 hpf) using an inverted confocal microscope (Zeiss LSM 780) with a × 40 water immersion objective (NA 1.2). Analysis was carried out as previously described (Ries et al., 2009; Yu et al., 2009). Measurements were taken from multiple embryos for each molecular pair, and the experiments were repeated at least twice. The number of embryos and total readings for each molecular pair are listed in Table 3. The plasmids used for generating various mRNA are listed in Table 5. The fusion constructs for sqt, cyc, and lefty have been previously described and used in FCCS experiments (Wang et al., 2016). For fgf8-RFP, the fgf8-GFP construct (Yu et al., 2009) was used as a template to replace the fluorescent protein.

**Table 5:** Plasmids used for FCCS

Reagent	Source
pCS2: squint-eGFP	Wang et al., 2016
pCS2: cyclops-eGFP	Wang et al., 2016
pCS2: lefty-mCherry	Wang et al., 2016
Pcs2: Ism1-eGFP	This study
Pcs2: Ism1-RFP	This study
Pcs2: Fgf8a-RFP	This study

### Quantitative Real-Time PCR (qRT PCR)

For qRT PCR analysis of *Ism1* and *Ism2* in Ism1^−/−^ mutants and wild type, first, tail biopsies for genotyping were taken from 24 hpf embryos and the rest of the embryo was utilized for RNA extraction. PCR genotyping was performed to identify homozygotes before proceeding with qRT PCR analysis.

Ism1-eGFP mRNA (200 pg) or the plasmid expressing Ism1-eGFP (25 pg) was injected into 1 cell stage wild type embryos. Embryos expressing strong eGFP were selected, 10 such embryos were pooled, and 3 such groups were collected. As control, mCerulean mRNA or plasmid was injected and processed identically as the Ism1-eGFP injected embryos. All embryos were collected at 50% epiboly (5.3 hpf), lysed in extrazol (BLIRT S.A.), and subjected to RNA extraction. All samples were treated with DNAse. One-step real time reverse transcription PCR (Takara) was performed to quantify expression of *ntl* and *sox32* in the Ism1-eGFP embryos, and their levels were compared with that in control embryos. Beta-actin was used as the house-keeping gene to normalize expression values. All qRT PCR primers are listed in Table 6. Fold changes were calculated using the 2^−ΔΔC^_T_ (Livak and Schmittgen, 2001) and the two tailed, unpaired “*t*” test was used to calculate statistical significance at a “*p*” value of 0.05 (GraphPad prism, ver. 5.0).

**Table 6:** List of primers for PCR genotyping and qRT PCR

Gene	Primer	Comments
*Ism1*_Genotyping_F1	ACGGAATCAAGCTAATAACCGAGA	F1 and R1 used for genotyping 55 bp deletion mutants
*Ism1*_Genotyping_R1	AGGGGAACTAGGCAAAGTCA	
*Ism1*_Genotyping_R2	GGTCCAGATGGATGTTGGCTC	F1 and R2 used for genotyping 2.5 Kb deletion mutants
*ntl*_qRT_F	GAACCACAGAGCTGCTCCATATC	
*ntl_*qRT_R	CTGGTGTTGGAGGTAGTGTTTGTG	
*Sox32*_qRT_F	GGACCTGGAGAACACTGACCT	
*Sox32*_qRT_R	CGGGGCCGGTATTTGTAGTTA	
*Ism1*_qRT_F	TACATAGACGGGGAAGGCGA	
*Ism1*_qRT_R	GTTCATCCAACGCTCACAGC	
*Ism2*_qRT_F	CGGCGTCTACGTTGAAAATCA	
*Ism2*_qRT_R	CAGGTTCTGGCAGTGGTAGG	
*betaActin*_qRT_F	GTGCCCATCTACGAGGGTTA	
*betaActin*_qRT_R	TCTCAGCTGTGGTGGTGAAG	

## Electronic supplementary material

Below is the link to the electronic supplementary material.

Supplementary file1 (DOCX 13.7 kb)
