# Supplementary material for: Isthmin1, a secreted signaling protein, acts downstream of diverse embryonic patterning centers in development
Source: Cell Tissue Res. 2020 Dec 28;383(3):987–1002. doi: 10.1007/s00441-020-03318-2 (PMC7960586; doi:10.1007/s00441-020-03318-2)
Supplement: Supplementary file 1 — Supplementary file1 (DOCX 13.7 kb) [file 441_2020_3318_MOESM1_ESM.docx]

Supplementary Figure S1

S1a: Injection of untagged GFP RNA leads to GFP localization in the cytoplasm (arrowhead) in 24hpf larva. (b) Injection of RNA for an Ism1-GFP fusion construct leads to accumulation of GFP in the ventricular fluid (asterisk), extracellular space, and vesicular structures (arrowheads in insert). There were no obvious developmental abnormalities in Ism1-GFP injected embryos. (c) Amino acid sequences of Ism1 from zebrafish (Danio rerio, Ensembl id: ENSDARG00000020541), chick (Gallus gallus, Ensembl id: ENSGALG00000009042) and mouse (Mus musculus; Ensembl id: ENSMUSG00000074766) were aligned using the multiple sequence alignment tool (clustal omega alignment tool; EMBL-EBI). Most sequence variations were observed in the N-terminus region with the protein domains showing higher conservation. SP (signal peptide), ISD (Isthmin Specific Domain), TSR (thrombospondin-type 1 repeat), and AMOP (an adhesion-associated domain in Muc4 and other proteins). (*) indicates identical residue, (:) indicates conserved substitution of residues, (.) indicates semi-conserved substitution and ( ) gap indicates absence of amino acid. Scale bar 20 μm.
